# Water quality and the CO_2_-carbonate system during the preconditioning of Pacific oyster (*Crassostrea gigas*) in a recirculating aquaculture system

**DOI:** 10.1038/s41598-022-26661-6

**Published:** 2022-12-23

**Authors:** Salvador Villasuso-Palomares, María T. Gutiérrez-Wing, Carmen G. Paniagua-Chávez

**Affiliations:** 1 Departamento de Acuicultura, Centro de Investigación Científica y de Educación Superior de Ensenada, Baja California (CICESE), Carretera Ensenada-Tijuana No. 3918, Zona Playitas, 22860 Ensenada, Baja California Mexico; 2 Louisiana State University Agricultural Center (LSU AgCenter), School of Renewable Natural Resources/Aquatic Germplasm and Genetic Resources Center, 2288 Gourrier Ave, Baton Rouge, LA 70820 USA

**Keywords:** Biological techniques, Biotechnology

## Abstract

The continued increase of the demand for seed of the Pacific oyster (*Crassostrea gigas*) has driven the aquaculture industry to produce land-based hatcheries using broodstock conditioning. This has led to the need to create closed systems to control the main factors involved in reproduction (temperature and food). Additionally, reproductive synchronization of broodstocks may be considered to ensure homogeneous maturation and spawning among the organisms. In this work, we synchronized the broodstock reproductive stage of Pacific oysters in a recirculating aquaculture system (RAS) using a “preconditioning” process and evaluated the effect of the water quality and the CO_2_-carbonate system on preconditioned broodstock. The oysters were kept at 12 °C for 45 days in a RAS containing a calcium reactor (C2) or without a calcium reactor (C1, control). Water quality parameters were measured daily, and the oyster’s condition and reproductive development were monitored using condition index, biometrics, and histology, on Days 0, 20, and 45. C1 and C2 systems kept the water quality within the ranges reported as favorable for bivalves. The calcium reactor kept the pH (8.03–8.10), alkalinity (200 mg/L as CaCO_3_), CO_3_^2−^ (≤ 80 µmol/kg), and Ω aragonite (≤ 1) closer to the ranges reported as optimal for bivalves. However, no significant differences were detected in the total weight and the condition index in C1 and C2. The preconditioning allowed to maintain the organisms in early reproductive development, allowing gametogenesis synchronization to start maturation.

## Introduction

Pacific oyster (*Crassostrea gigas*) is the most cultured bivalve worldwide^1^. However, *C. gigas* production faces a significant challenge: continuous seed production to meet current demands^2^. Consequently, the conditioning of oyster broodstocks in hatcheries to produce high-quality seeds is an important issue. Broodstock conditioning is a process by which an organism’s reproductive cycle is controlled by manipulating the main factors involved as the temperature or the feeding rates^3^. However, the onset of the initial reproductive stage in wild broodstocks is variable among organisms, and it is not possible to verify at least the organisms are open. Thus, the reproductive stage of an organism is uncertain at the beginning of conditioning, and the time required to produce mature gametes among the oysters constantly fluctuates^4^. Therefore, the objective of preconditioning is to induce all organisms to reach maturation at the same time.

We define preconditioning as the process of synchronizing a broodstock's reproductive stage by manipulating the biological zero temperature (temperature below which reproductive development does not occur^3^) to initiate maturation at the same reproductive stage. The maintenance of a preconditioned broodstock will allows for staggered larval production throughout the year. The needs of oyster farmers can be better served with a continuous, timely supply of high-quality larvae.

Recirculating aquaculture systems (RASs) can be of benefit to broodstock reproductive conditioning, as RASs can maintain optimal water quality for prolonged periods and an accurate control of the factors involved in the reproductive cycle of *C. gigas*^5^. However, it is vital to know the performance of an RAS because it may cause water acidification, which is produced by the nitrification process and by the hydrolysis of CO_2_ from the respiration of the organisms and of nitrifying bacteria. The final result of this biochemical process is the modification of pH, alkalinity, the carbonate (CO_3_^2−^) concentration, the bicarbonate (HCO_3_^−^) concentration, and the saturation state of calcite and aragonite (the CO_2_-carbonate system)^6,7^.

Control over the CO_2_-carbonate system in an RAS is critical, particularly when used for bivalve mollusks, because it can affect the acid–base balance, influence biomineralization-related processes, increase shell dissolution, change the metabolic rate, shift energy budgets, and decrease survival^8^. Consequently, the organisms could expend energy mitigating stress-inducing conditions instead of storing energy (as glycogen) or producing gametes^9^. Thus, the objective of this work was to evaluate the effects of water quality and the CO_2_-carbonate system on broodstock preconditioning and to synchronize gametogenesis of a Pacific oyster (*Crassostrea gigas*) broodstock in an RAS.

## Materials and methods

The oysters were kept under their biological zero condition (12 °C) for 45 days. The organisms were distributed under two experimental conditions: RAS with no control over the CO_2_-carbonate system (C1) and RAS with a CO_2_-carbonate system that was controlled through a calcium reactor (C2).

### Organisms

One-year-old *Crassostrea gigas* oysters (822 organisms), cultured in Guerrero Negro, B. C., Mexico (27°57′32.20″ N; 114°3′23.95″ W), were donated by the company Acuacultura Integral de Baja California S. A. de C.V. The organisms were transported to the Department of Aquaculture at CICESE. Upon arrival, the organisms were individually examined and cleaned. Epibionts, organic matter, and mussel seeds were removed from the shells. Subsequently, ten oysters were used to determine the condition index, and fifteen were used to determine reproductive development. For preconditioning, the remaining 796 oysters were distributed between the two experimental conditions, C1 and C2.

### Recirculating aquaculture systems (RAS)

The experimental system for each condition (C1 and C2) consisted of one RAS with a culture tank containing 1.3 m^3^ seawater. A moving bed biofilter, which contained 110 L of Kaldnes™ K1 media (specific surface area: 500 m^2^/m^3^) to support nitrifying bacteria, was used for nitrification and degassing. A 100-L plastic container with 20 L of an elliptical polyethylene floating filter consisting of bead media AB1 (Pentair Aquatic Eco-System) was used for solid waste removal. The water was circulated at a rate of 57 L/min using a magnetic pump (Little Giant Model 3-MD-SC, Oklahoma City). The water temperature was controlled with an air-cooled heat pump (Delta Star^®^, Aqua Logic DSHP-5) and was adjusted to 12 °C before replacement to avoid thermal shock in the organisms. A weekly 500-L water replacement was performed on each RAS to remove solids.

#### Calcium reactor (CA)

A calcium reactor was used to control the CO_2_-carbonate system. The calcium reactor's purpose was to constantly add CO_3_^2−^ ions to buffer H^+^ produced from nitrification and CO_2_ hydrolysis. For Condition C2, the calcium reactor was connected to the biofilter. The calcium reactor consisted of a reaction chamber (40 cm high × 10.16 cm diameter) connected by a hose (0.635 cm diameter) to a counter bubble chamber (16.5 cm high × 5.2 cm diameter), and a Quiet One^®^ 1200 magnetic pump (Lifergard Aquatics) was used for water circulation. The reaction chamber contained 1 kg of a crushed coral-based calcium reactor medium (ReBorn™, Florida, US). Coral-based media dissociation was increased by CO_2_ (1.3–1.5 mL/min) injection through the counter bubble chamber. The calcium reactor design was described by Sanjay Joshi^10^.

### Oyster preconditioning

Once the organisms were distributed across the two experimental conditions, the oysters were kept below their biological zero condition (12 °C) for 45 days and fed daily with 20 mL (2 × 10^9^ cells/mL) of microalgae concentrate (Shellfish diet 1800™, Reed Mariculture). This commercial concentrate contained a mixture of six different microalgae: *Isochrysis* sp.*, Pavlova* sp., *Tetraselmis* sp.,* Chaetoceros calcitrans*, *Thalassiosira weissflogii*, and *Thalassiosira pseudonana.* The final microalgae concentration per RAS was 27 × 10^3^ cells/mL.

Twenty oysters were sampled on Days 20 and 45 to determine their total weight, condition index, and reproductive development.

### Water quality

Water quality was measured daily for both experimental conditions. Temperature, salinity, and dissolved oxygen (DO) were measured with a Pro Dss multiparameter meter (YSI^®^; Ohio, US). The total ammonia nitrogen (TAN) was determined using Solorzano's indophenol method^11^. Nitrite nitrogen (NO_2_-N) was determined with a method described by Shin and modified by Bendschneider and Robinson^12^ for seawater. Nitrite nitrogen was measured using sulfanilamide hydrochloride and *N-*(*1*-naphthy1)-ethylenediamine dihydrochloride to form an azo dye, which was measured at a wavelength of 543 nm. Nitrate nitrogen (NO_3_-N) was determined by an ultraviolet spectrophotometric screening method according to a methodology described in Standard Methods for the examination of water and wastewater^13^. TAN, NO_2_-N, and NO_3_-N measurements were made using an Epoch™ spectrophotometer (Biotek^®^ Instruments, Winooski, US). All nitrogen compound measurements were carried out in triplicate.

### CO_2_-carbonate system

The alkalinity was determined with a colorimetric method described by Adams (1990), where aliquots of 10 mL were titrated with 0.02 N H_2_SO_2_^14^. The pH was measured with a Pro Dss multiparameter (YSI^®^; Ohio, US) on an NBS scale (US National Bureau of Standards). The partial pressure of CO_2_ (pCO_2_), bicarbonate (HCO_3_^−^) concentration, carbonate (CO_3_^2−^) concentration, and the saturation states of calcite (Ω_ca_) and aragonite (Ω_ar_) were calculated according to measured values of pH, alkalinity, salinity, and temperature using the dissociation constants described by Mehrbach et al.^15^ with a software CO_2_ System (CO_2_ sys) by Lewis and Wallace^16^.

### Survival, total weight, and condition index (CI)

Survival was monitored throughout the experiment, and the survival rate under both the C1 and C2 experimental conditions was recorded. The same oysters were used to measure the total weight and determine the condition index (CI). The CI was calculated according to an equation described by Hickman and Illingworth^17^

$${\text{CI}} = \frac{{\text{dry weight of the soft tissue}}}{{\text{total weight}}-{\text{shell weight }}} \times 100.$$

### Reproductive development

Tissue samples (4-mm wide slices) obtained from a location between the labial palps and gills of the 20 oysters in each sample were fixed in Davidson's solution for 48 h^18^. After fixation, the samples were dehydrated in ascending concentrations of ethanol solutions and embedded in paraffin wax. Then, 5-µm thick slices were stained with hematoxylin and eosin and mounted on glass slides^18^. The slides were observed with an Olympus microscope (CX31RTSF) at 400 ×, and reproductive development was determined using the seven-stage reproductive scale (undifferentiated, developing -early active, developing-late active, ripe, partially spent, totally spent, postspawning, and resorption) described by Steele and Mulcahy^19^.

### Data analysis

The total weight and CI were tested using a Mann–Whitney *U* test on Days 20 and 45 for C1 and C2. A value of *P* < 0.05 was chosen as the level of significance. Statistical analysis was performed using Statistica 7.1 (Stat Soft, Inc.).

### Ethical approval and consent to participate

No approval from research ethics committees was required because the experimental work was conducted with an unregulated invertebrate species.

## Results

### Water quality

The temperatures for C1 and C2 were maintained at biological zero (12 °C) during experimentation. The mean temperature during the 45 days of experimentation was 11.34 °C ± 0.39 °C for C1 and 11.56 °C ± 0.55 °C for C2. The mean DO concentrations under both experimental conditions were 8.00 mg/L ± 0.10 in C1 and 7.99 mg/L ± 0.14 in C2. The salinity was 34.02 ± 0.08 ppt in C1 and 33.81 ± 0.07 ppt in C2 (Table 1).

**Table 1:** Water quality factors for two experimental conditions (EC) in the pre-conditioning of the Pacific oyster *C. gigas*. *C1* recirculating aquaculture system (RAS) without calcium reactor (control), *C2* RAS with the calcium reactor, *DO* dissolved oxygen, *TAN* total ammonia nitrogen, *N-NO*_*2*_ nitrogen nitrites, *N-NO*_*3*_ nitrogen nitrates.

EC	Temperature (°C)	DO (mg/L)	Salinity (ppt)	TAN (mg/L)	N-NO_2_ (mg/L)	N-NO_3_ (mg/L)
C1	11.34 ± 0.39	8.00 ± 0.10	34.02 ± 0.08	1.26 ± 0.52	0.08 ± 0.04	46.88 ± 25.08
C2	11.56 ± 0.55	7.99 ± 0.14	33.81 ± 0.07	1.42 ± 0.64	0.20 ± 0.08	38.15 ± 14.67

The mean concentration of TAN for both experimental conditions was > 1 mg/L (1.26 ± 0.52 for C1 and 1.42 ± 0.64 for C2). The nitrite nitrogen concentration was higher in C2 (0.20 ± 0.08 mg/L) compared with the concentration for C1 (0.08 ± 0.04 mg/L). The mean nitrate nitrogen concentration was 46.88 ± 25.08 mg/L in C1 and 38.15 ± 14.67 mg/L in C2 (Table 1).

### CO_2_-carbonate system

The RAS with the calcium reactor (C2) showed a higher buffer capacity, with a mean alkalinity of 147 ± 16 mg/L CaCO_3_ compared with 107 ± 5 mg/L CaCO_3_ for C1 (Table 2). The pH under both experimental conditions was < 8.0 during preconditioning. The control treatment had a lower pH value (7.77 ± 0.07) than C2 (7.88 ± 0.05).

**Table 2:** CO_2_-carbonate system factors for two experimental conditions (EC) in the pre-conditioning of the Pacific oyster *C. gigas*. *C1* recirculating aquaculture system (RAS) without calcium reactor (control), *C2* RAS with the calcium reactor, pH, *Alk* alkalinity, *pCO*_*2*_ partial pressure of carbon dioxide, *HCO*_*3*_^−^ bicarbonate, *CO*_*3*_^*2*−^ carbonate, *Ω*_*ar*_ saturation state of aragonite, *Ω*_*ca*_ saturation state of calcite.

EC	pH	Alk (mg/L CaCO_3_)	*p*CO_2_ (µatm)	HCO_3_^−^ (µmol/kg)	CO_3_^2−^ (µmol/kg)	Ω_ar_	Ω_ca_
C1	7.77 ± 0.07	107 ± 5	982 ± 191	1942 ± 104	57 ± 7	0.86 ± 0.11	1.36 ± 0.17
C2	7.88 ± 0.05	147 ± 16	1030 ± 185	2630 ± 304	98 ± 13	1.50 ± 0.20	2.36 ± 0.32

After 45 days of experimentation, the mean pCO_2_ was 982 ± 191 µatm for C1 and 1030 ± 185 µatm for C2. The mean HCO_3_^−^ concentration in C2 (2630 ± 304 µmol/kg) was higher compared with C1 (1942 ± 104 µmol/kg). The CO_3_^2−^ concentration of 98 ± 13 µmol/kg for C2 was higher compared with the concentration of 57 ± 7 µmol/kg for C1. The Ω_ar_ was < 1 in C1 and > 1 in C2. The Ω_ca_ was > 1 under both experimental conditions. Nevertheless, the mean concentration of Ω_ca_ after 45 days of experimentation was 2.36 ± 0.32 for C2 and 1.36 ± 0.17 for C1 (Table 2).

### Survival, total weight, and condition index (CI)

**Figure 1:**
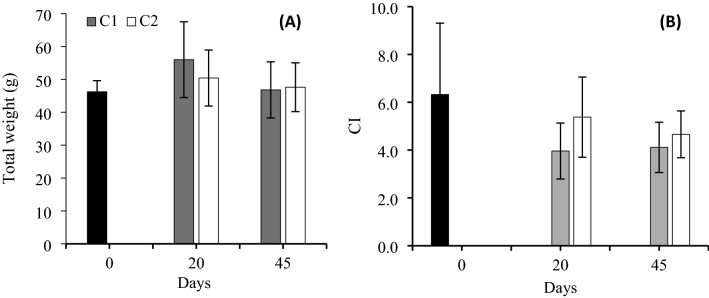
Total weight and condition indices (CI) during 45 days of pre-conditioning of the Pacific oyster *C. gigas* in two experimental conditions. Black bars, day 0; Grey bars C1, recirculating aquaculture system (RAS) without calcium reactor (control), and white bars C2, RAS with the calcium reactor. Biometry and CI were evaluated on days 0, 20, and 45. (**A**) Total weight (g); (**B**) Condition index. The error bars represent the standard deviation from the mean.

The total survival after 45 days of experimentation was 83.21% for C1 and 82% for C2. The total individual weight was 46.2 ± 3.4 g at the beginning of the experiment (Day 0). At the end of the experiment, the total individual weight for oysters in C1 was 46.8 ± 8.5 g and 47.6 ± 7.4 g in C2 (Fig. 1A). No significant differences were detected in the total weight on Day 20 (*P* = 0.28) or on Day 45 (*P* = 0.82) between C1 and C2. The oysters in C1 were significantly heavier (*P* = 0.28) on Day 20 than on Day 45. For C2, no significant differences (*P* = 0.40) were detected in the total weight between Days 20 and 45. No significant differences were detected in the total weight between Days 0 and 45 in oysters from C1 (*P* = 0.93) and C2 (*P* = 0.93).

The condition index on Day 0 was 6.3 ± 2.99 and reached 4.1 ± 1.05 in C1 and 4.7 ± 0.98 in C2 (Fig. 1B). Significant differences were detected in CI on Day 20 between C1 and C2 (*P* = 0.041). No significant differences were detected in CI on Day 45 between C1 and C2 (*P* = 0.15).

### Reproductive development

**Figure 2:**
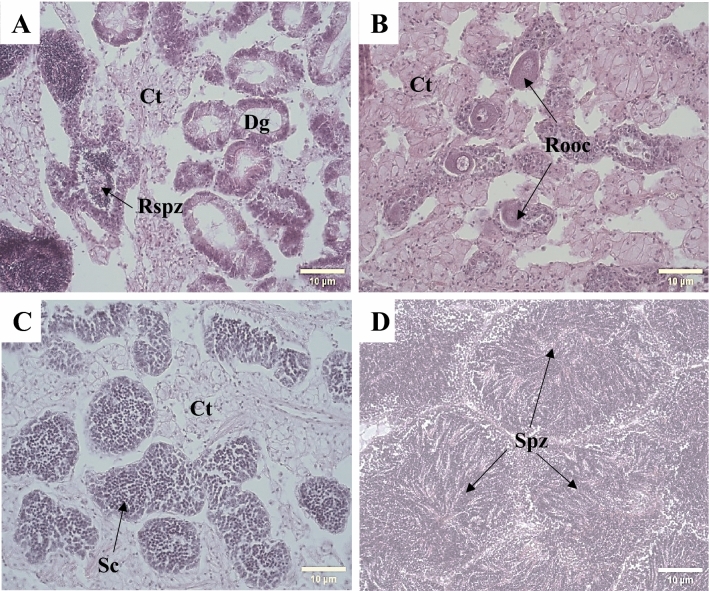
Histological description of the Pacific oyster *C. gigas* during 45 days of pre-conditioning in two experimental conditions. (**A**) Male in resorption stage (400 ×); (**B**) female in resorption stage (400 ×); (**C**) male in developing-early active stage (400 ×); (**D**) ripe male (400 ×); *Ct* conjunctive tissue, *Dg* digestive gland, *Rspz* residual spermatozoa, *Rooc* residual oocyte, *Sc* spermatocytes, *Spz* spermatozoa. Scale bar: 10 µm.

**Figure 3:**
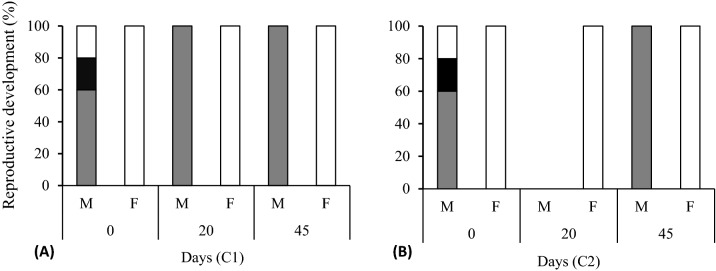
Reproductive development of the Pacific oyster *C. gigas* during 45 days of pre-conditioning in two experimental conditions. (**A**) Recirculating aquaculture system (RAS) without calcium reactor (control), C1, and (**B**) RAS with the calcium reactor, C2. Black bars, ripe; gray bars, developing-early active, and white bars, resorption. *M* male, and *F* female. Reproduction development was evaluated on days 0, 20, and 45.

Males and females were observed in all samples except on Day 20 for C2 (Fig. 2A,B). At the beginning of the experiment, 100% of the females and 20% of the males were in the resorption stage. Additionally, 60% of the males were in the developing-early active stage, and only 20% of the males were ripe (Fig. 2C,D). For Days 20 and 45, all females were in the resorption stage, and all the males were in the developing-early active stage for both experimental conditions (Fig. 3A,B).

## Discussion

We define preconditioning as a process that synchronizes the reproductive stage of an oyster’s previous broodstock conditioning. Preconditioning could become a helpful process for achieving predictable conditioning times. To achieve successful preconditioning, it is necessary to keep the temperature at biological zero to avoid initiating the oyster reproductive cycles. Additionally, it is necessary to keep the water quality and CO_2_-carbonate system at favorable ranges to avoid long-term exposure to stress-inducing conditions. The exposition of bivalves to unfavorable environmental conditions during their reproductive cycle could promote the lysis of gametes as a strategy to recycle energy to satisfy the increase in basal metabolism^19^.

The temperature (< 12 °C), salinity (~ 34 ppt), and OD (> 7.0 mg/L) after 45 days of experimentation were kept within optimal ranges to allow for reproductive development delay^20,21^. Additionally, food supply was not a limiting factor since it was maintained at a recommended concentration (100 × 10^6^ cells per oyster per day) to ensure proper nutrition of *C. gigas* at low temperatures and outside the reproductive cycle^22^.

The TAN concentration under both experimental conditions was in the ranges reported by Buchanan et al.^20^ (0.10 mg/L and 4.2 mg/L) for oyster conditioning in an RAS for eight weeks, with no negative effects. Under our experimental conditions, the nitrite nitrogen concentration was within the range recommended by Stone and Thomforde^23^ for aquatic organisms (0.05–0.5 mg/L). Therefore, we can conclude that the concentrations of TAN and N-NO_2_ under both experimental conditions (C1 and C2) did not significantly affect the oysters.

A controlled CO_2_-carbonate system is essential for oyster preconditioning in RASs because of the complex interactions of alkalinity, pH, pCO_2_, CO_3_^2−^, Ω_ar_, and Ω_ca_, with the nitrifying bacteria from the biofilter and with the oysters. During the nitrification process, nitrifying bacteria consume HCO_3_^−^ as an inorganic carbon source and produce H^+^ as a metabolic byproduct^6^. The constant addition of H^+^ to the water and consumption of HCO_3_^−^ reduces the pH and alkalinity, which consists of 90% HCO_3_^−^, promoting water acidification. However, CO_2_ accumulation can also affect pH and alkalinity.

When CO_2_ interacts with water, it produces carbonic acid (H_2_CO_3_), which decreases pH and alkalinity. H_2_CO_3_ can dissociate in H^+^ and HCO_3_^−^ or interact with CO_3_^2−^ to produce 2HCO_3_^−^. Then, H_2_CO_3_ reduces the concentration of CO_3_^2-^ and the saturation state of Ω_ar_ and Ω_ca_^24^. Therefore, the increase in H^+^ from nitrification and CO_2_ accumulation reduces the buffer capacity of the water, promoting corrosive conditions for the oysters. Long-term exposure to corrosive conditions can negatively affect the acid–base balance, somatic growth, and condition index, which increases shell dissolution, energy requirements, and mortality^25^.

Alkalinity determines the acid-neutralizing capacity of water. For C1, the alkalinity was lower since C1 did not have an external source of CO_3_^2−^, while C2 had a calcium reactor. For RAS, an alkalinity of 200 mg/L CaCO_3_ is recommended for the proper operation of biofilters^26^. However, the low alkalinity did not impact biofiltration performance, as the nitrogen compound concentrations were kept within the optimal ranges for both experimental conditions.

The pH obtained under both experimental conditions was < 8.0 due to the nitrification process and due to the hydrolysis of CO_2_^6,7^. The pH in C1 and C2 differed from those reported to be optimum for *C. gigas*, between 8.03 and 8.10^8,23^. Nevertheless, Boulais et al.^27^ tested a pH of 7.5 on the gametogenesis of *C. virginica* and did not find any significant effects. Similarly, Lannig et al.^28^ did not find significant differences in the standard metabolic rates at a pH of 8.07 and at a pH of 7.68 at 15 °C for *C. gigas*. Therefore, in our study, the pCO_2_ was not high enough and the pH was not low enough to negatively affect the oysters.

In our experiment, the pCO_2_ under both experimental conditions was higher than those reported as normal conditions for bivalves (374–658 µatm) but lower than values at which adverse effects have been reported (2170–2625 µatm)^8,28–32^.

The CO_3_^2−^ concentration for C1 was below the threshold of 80 μmol/kg, which can promote a drastic decline in calcification^7^. Additionally, Ω_ar_ was > 1, and Ω_ca_ was slightly higher than 1, which could affect the calcification rates and shell dissolution rates to compensate for the saturation state of Ω_ar_ and Ω_ca_^33^. According to our results, a calcium reactor helps maintain pH, alkalinity, HCO_3_^−^, CO_3_^2−^, Ω_ar_, and Ω_ca_ at values close to the optimal ranges. However, no significant differences were detected in total weight or CI between C1 and C2 after 45 days of experimentation.

The water quality factors, alkalinity, pH, and pCO_2_ under both experimental conditions were within ranges that are reported to be favorable to *C. gigas*, so the RASs probed to be reliable providing optimal conditions for the preconditioning. Despite the relatively low CO_3_^2−^ concentration and Ω_ar_ saturation state in C1, no differences were observed in the CI or the total weight of the oysters after 45 days of experimentation. Therefore, using a calcium reactor may not be necessary during preconditioning with the loading and times tested.

The temperature is the most important factor involved in the reproductive cycle of *C. gigas* because can accelerate or delay reproductive development^4^, so it was vital to maintain the 12 °C during preconditioning to avoid the initiation of gametes maturation. For *C. gigas* has been reported when organisms are maintained below the minimum spawning temperature, the resorption process could initiate, which is characterized by the cleaning of the mature gametes so that the gonad can prepare for its next reproductive cycle^19^. Thus, the preconditioning temperature allowed male oysters in resorption and ripe stages could change to an early reproductive development stage. In addition, several authors have reported a minimum spawning temperature of ~ 18 °C for *C. gigas*^19,34^. The temperatures in C1 and C2 were never higher than 12 °C. Therefore, we can conclude that gametogenesis synchronization of *C. gigas* under both experimental conditions was due to the preconditioning process and not due to the complete or partial spawning of the oysters.

The results for oyster reproductive development under both experimental conditions showed that 12 °C is a favorable temperature for *C. gigas* to allow for gametogenesis synchronization and is low enough to avoid gamete maturation. Additionally, the results showed that 45 days of preconditioning could be enough to promote gametogenesis synchronization in *C. gigas*. Also, the results showed that the calcium reactor has no significant effect on the gametogenesis synchronization in *C. gigas*. However, further investigations are necessary to evaluate gametogenesis synchronization for longer times, if preconditioning enhances the maturation process during broodstock conditioning and the effect of preconditioning in more than one reproductive cycle.

## Data Availability

The datasets generated in the current study are available from the corresponding author upon reasonable request.
